# Multitarget Therapeutic Strategies for Alzheimer's Disease: Review on Emerging Target Combinations

**DOI:** 10.1155/2020/5120230

**Published:** 2020-06-30

**Authors:** Samuele Maramai, Mohamed Benchekroun, Moustafa T. Gabr, Samir Yahiaoui

**Affiliations:** ^1^Department of Biotechnology, Chemistry and Pharmacy, Department of Excellence 2018−2022, University of Siena, Via Aldo Moro 2, 53100 Siena, Italy; ^2^Conservatoire National des Arts et Métiers, Équipe de Chimie Moléculaire, Laboratoire de Génomique Bioinformatique et Chimie Moléculaire, GBCM, EA7528, 2 Rue Conté 75003 Paris, France; ^3^Department of Radiology, Stanford University School of Medicine, Stanford, CA 94305, USA; ^4^Department of Drug Design and Optimization, Helmholtz Institute for Pharmaceutical Research Saarland, Campus E8.1, 66123 Saarbrücken, Germany

## Abstract

Neurodegenerative diseases represent nowadays one of the major health problems. Despite the efforts made to unveil the mechanism leading to neurodegeneration, it is still not entirely clear what triggers this phenomenon and what allows its progression. Nevertheless, it is accepted that neurodegeneration is a consequence of several detrimental processes, such as protein aggregation, oxidative stress, and neuroinflammation, finally resulting in the loss of neuronal functions. Starting from these evidences, there has been a wide search for novel agents able to address more than a single event at the same time, the so-called multitarget-directed ligands (MTDLs). These compounds originated from the combination of different pharmacophoric elements which endowed them with the ability to interfere with different enzymatic and/or receptor systems, or to exert neuroprotective effects by modulating proteins and metal homeostasis. MTDLs have been the focus of the latest strategies to discover a new treatment for Alzheimer's disease (AD), which is considered the most common form of dementia characterized by neurodegeneration and cognitive dysfunctions. This review is aimed at collecting the latest and most interesting target combinations for the treatment of AD, with a detailed discussion on new agents with favorable *in vitro* properties and on optimized structures that have already been assessed *in vivo* in animal models of dementia.

## 1. Introduction

Neurodegeneration is a pathological process that causes the progressive loss of neuronal function and leads to cognitive impairments, memory loss, and several forms of ataxia. This feature is pivotal in illnesses such as Alzheimer's disease (AD), Parkinson's disease (PD), Huntington's disease (HD), or Amyotrophic Lateral Sclerosis (ALS). Neurodegenerative diseases represent a heavy economic and social threat for our societies, especially now in low-to-middle income countries. According to the World Health Organization, around 50 million people—mostly elderly—are affected by dementia with AD representing *ca.* 60-70% of the cases [1]. Given the global increase in life expectancy, prodigious efforts have to be made to find new neuroprotective medicines able to impede, or even reverse, the neurodegeneration.

From a biochemistry perspective, neurodegenerative diseases share between them common pathological processes such as protein misfolding and aggregation, altered levels of neurotransmitters (e.g., acetylcholine and dopamine), metal ion dyshomeostasis [2], mitochondrial malfunction, oxidative stress, and neuroinflammation [3].

For instance, in AD, abnormal histological changes are characterized by the deposition of *β*-amyloid (A*β*) plaques formed out of aggregated A*β* fibrils and neurofibrillary tangles (NFTs) made of hyperphosphorylated TAU protein (pTAU) [4]. PD proteopathy is linked to misfolded aggregates of *α*-synuclein (*α*-syn) accumulated in Lewis bodies [5]. In ALS, histological studies have shown the presence of aggregates of mutant superoxide dismutase 1 (SOD1), TAR DNA binding protein (TDP-43), fused in sarcoma (FUS), and repeat dipeptides from noncanonical translation of mutant chromosome 9 open reading frame 72 (C9ORF72) [6].

**Figure 1:**
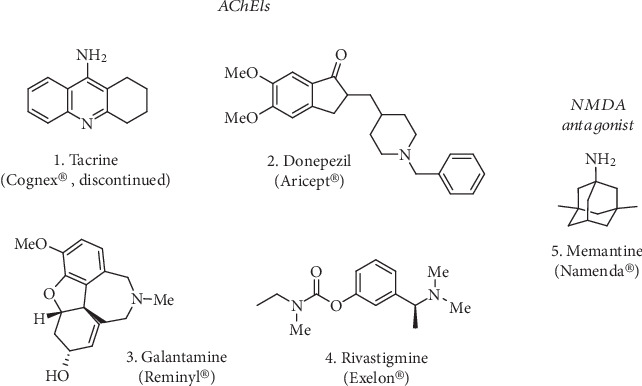
AChE inhibitors marketed for the treatment of AD (1-4) together with the NMDA receptor antagonist Memantine (**5**).

The other main pathological event leading to neurodegeneration is oxidative stress. Even if the human brain constitutes only 2% of the body mass, it consumes 20% of the oxygen brought by the respiratory system [7]. This feature renders the brain more vulnerable towards oxidative stress. Thus, oxidation of the main constituents of neurons (lipids, proteins, and nucleic acids) leads invariably to neurodegeneration [8]. In other words, the constant accumulation of reactive oxygen and nitrogen species (ROS and RNS) leads to the ineluctable damage to neurons. This oxidative stress is caused by various underlying factors such as mitochondrial dysfunction [9, 10], dyshomeostasis of metal ions (e.g., redox-active Fe^2+^/Fe^3+^ and Cu^+^/Cu^2+^) and their role in promoting the deposit of aggregation-prone peptides (e.g., A*β* and *α*-syn) [11–13], and neuroinflammation [14, 15]. There is a global consensus on the fact that these etiologic mechanisms coexist simultaneously, influencing each other at multiple levels [16]. Consequently, these pathological features are responsible for neuronal cell death and dysfunction in neurotransmission translating into progressive cognitive impairment and/or ataxia. Based on their intertwined roles in the etiology of neurodegenerative diseases, they represent crucial therapeutic targets. Current treatments available in the market for neurodegenerative diseases are mainly palliative and poorly ameliorate the day-to-day life of patients. For instance, the treatments available now in the market for AD consists of three inhibitors of acetylcholinesterase (AChEIs, Figure 1), which maintain the levels of acetylcholine (ACh) and thus the neurotransmission [17]; along with Donepezil, Galantamine, and Rivastigmine (**2**-**4**, Figure 1) approved for mild-to-moderate AD, one NMDA antagonist, Memantine (**5**, Figure 1), has been approved for moderate-to-severe AD [18]. Tacrine (THA, **1**, Figure 1) was the first AChEI to be marketed for AD treatment but was rapidly discontinued due to its hepatotoxicity [19].

**Figure 2:**
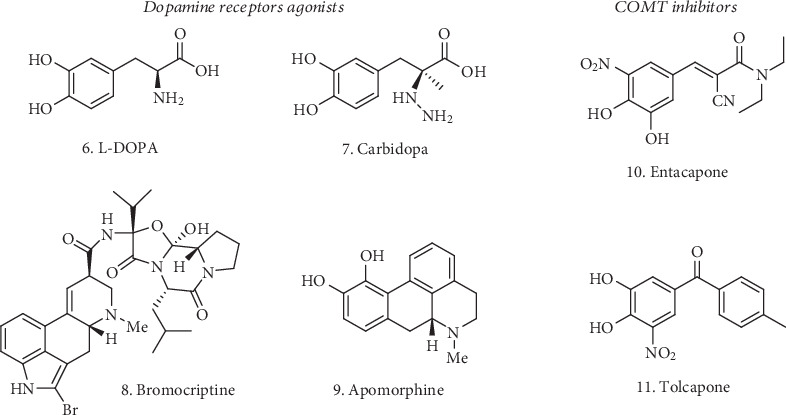
Representative dopamine receptor agonists (**6-9**) and COMT inhibitors (**10** and **11**) for the treatment of PD.

Available treatments for PD consist mainly in restoring dopaminergic tone either by administering catecholamines such as L-DOPA and Carbidopa (**6** and **7**, Figure 2) or dopaminergic receptor agonists such as ergot-derived alkaloids (bromocriptine, apomorphine (**8** and **9**, Figure 2), cabergoline, lisuride, and pergolide) and non-ergot-derived small-molecules (pramipexole, ropinirole, and piribedil). Because of the short half-life of L-DOPA, catechol-*O*-methyltransferase (COMT) inhibitors (e.g., Entacapone and Tolcapone (**10** and **11** respectively, Figure 2) are often coadministered with L-DOPA to block COMT-mediated metabolism, thus maintaining a longer dopaminergic tone.

**Figure 3:**
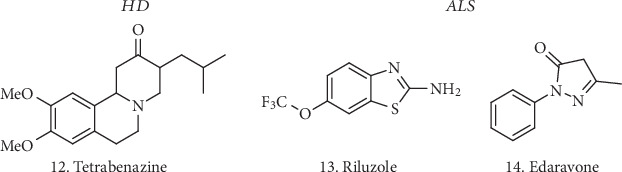
Compounds used in the treatment of hyperkinetic movement disorders related to HD (**12**) and used to slow down the progression of ALS (**13** and **14**).

Concerning HD, there is no treatment available to alter the course of the disease. However, there are medications able to lessen movement disorders such as Tetrabenazine (**12**, Figure 3). Antipsychotics, antidepressants, and tranquilizers might be also used. ALS treatments include only palliative drugs such as Riluzole and Edaravone (**13** and **14**, Figure 3) that bring serious side effects such as dizziness and headache, as well as gastrointestinal and liver problems.

Neurodegenerative diseases have a highly intricate etiology where many biological factors concur simultaneously at various levels to induce the neurodegeneration. This critical aspect represents a veritable hurdle for the development of disease-modifying drugs able to target the profound causes of neurodegeneration. The failure of “one drug-one target” drug design strategy and the multifunctional nature of neurodegenerative diseases inspired the scientific community to investigate the effectiveness of another drug design strategy called “designed multiple ligands,” “hybrid molecules,” or “multitarget-directed ligands” (MTDLs). This emerging strategy is centered on the development of pleiotropic ligands able to interact at least with two therapeutic targets at the same time. The idea of MTDLs has been largely pursued for the discovery of a more efficacious treatment for AD, and a great amount of structures based on this polypharmacology concept have been proposed [20]. Some of the most appealing analogues are the result of molecular hybridization, where the combination of multiple pharmacophores should reproduce the activity of the parent compounds while retaining a certain degree of selectivity towards the selected targets. These hybrid structures can be combined (*i*) by using a linker that spaces and anchors the biologically active moieties, (*ii*) by fusing the active sections together, (*iii*) or simply by merging the functionalities known to be involved in the target engagement [21]. The rational design behind these potential new drugs has been frequently inspired by well-known and/or approved drugs such as THA [22, 23], Donepezil [24, 25], or Rivastigmine, along with different natural bioactive derivatives such as resveratrol or curcumin [26], although other very interesting structural combinations/modifications have been recently identified. Here, we report the most recent and more interesting examples of newly developed MTDLs which are able to interact and modulate different biological systems and represent potential prototypes for a new treatment of AD.

## 2. Target Combinations in MTDL Design Strategy for AD

The cholinergic deficit represents an undeniable cause of AD. ACh plays a pivotal role in cognitive processes, and disruptions in its neurotransmission can influence all the aspects of cognition and behavior, not only in AD but also in other age-related forms of dementia [27]. Acetylcholinesterase (AChE) rapidly terminates the action of ACh in the synaptic cleft, leaving choline and acetate as the products of its hydrolytic activity. Butyrylcholineterase (BuChE) also plays an important role in cholinergic mediation [28].

Cholinesterases (ChEs) inhibitors can increase the levels of ACh and contribute to upregulate the cholinergic tone in neurons, partially ameliorating cognitive symptoms. AChE is a particularly attractive target to address AD-related symptoms, not only for its catalytic functions but also for the effects on A*β* precipitation, plaque formation [29], and inflammation. As discussed before, a few compounds have made their appearance in the market, like Donepezil or Rivastigmine (**2** and **4**, Figure 1), which were approved for the treatment of mild-to-moderate AD symptoms.

In addition to the cholinergic deficit, the presence of extracellular A*β* peptide plaques and NFTs of hyperphosphorylated pTAU represent the other main pathological features. Therefore, the “amyloid cascade hypothesis” is still the main focus for AD treatment. A*β* is generated from the Amyloid Precursor Protein (APP) by sequential cleavages, involving the *β*-secretase beta-site APP-cleaving enzyme 1 (BACE-1) in the rate limiting step. Over the years, a great variety of BACE-1 inhibitors have been proposed and have entered clinical trials, highlighting the central role of this protease in AD [30].

**Figure 4:**
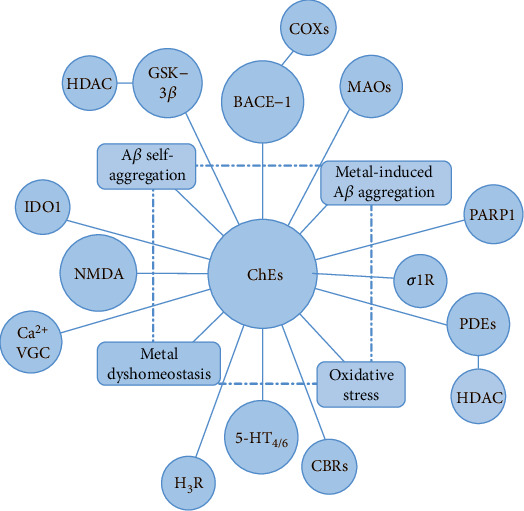
Schematic representation of some recent and interesting combinations regarding ChEs and BACE-1 inhibitors and/or other targets involved in AD. Beside the dual action on the selected systems, most of the newly developed analogues have the potential to affect A*β* peptide aggregation along with oxidative stress and metal dyshomeostasis.

The combination of ChEs and/or BACE-1 inhibition with the activity on additional enzymatic/receptor systems and the effects on other AD-related alterations, such as metal dyshomeostasis and oxidative stress (Figure 4), opens up the way for the identification of very interesting MTDLs and represents the preferred approach for the discovery of new treatments.

### 2.1. Dual ChE/BACE-1 Inhibitors

As mentioned above, ChEs and BACE-1 are pivotal targets for AD. A sensible approach for the discovery of new MTDLs may be represented by the concomitant inhibition of these two enzymatic systems. To this aim, a good variety of structures has been presented.

**Figure 5:**
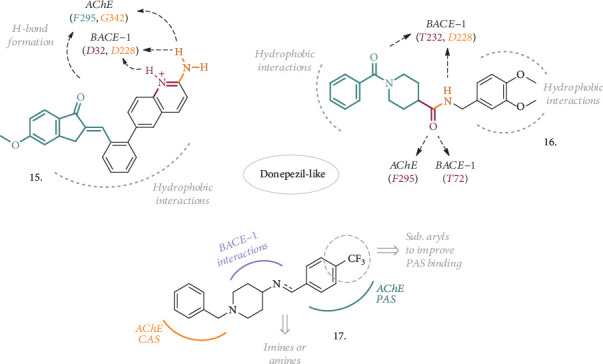
Structures of inspired- or rigid analogues of Donepezil as dual AChE and BACE-1 inhibitors. Highlighted are the main interactions with the two enzyme residues, mainly represented by H-bonds and hydrophobic interactions.

Gabr and Abdel-Raziq recently explored rigid analogues of Donepezil for their double activity against AChE and BACE-1 (**15** and **16**, Figure 5). Compound **15** [31] is a combination of features from Donepezil and other BACE-1 inhibitors, such as AZD3839 [32] or the simple 2-aminoquinoline ring, which shares with the AZD compound the bidentate interaction in the enzyme active site. The further addition of a double bond to connect the indenone moiety to the rest of the molecule afforded a nanomolar inhibitor of AChE and BACE-1, with IC_50_ = 14.7 nM and 13.1 nM, respectively. Kinetic studies on AChE revealed a concentration-dependent mixed-type inhibition of this enzyme, while the improved activity on BACE-1 confirmed the pivotal role played by the aminoquinoline group. The viability of SH-SY5Y neuroblastoma cells was not affected by concentrations of up to 50 *μ*M. Moreover, the compound 15 had the potential to be brain penetrant, showing high permeability in the PAMPA-BBB assay, and had considerable metabolic stability in rat liver microsomes.

Another interesting series of analogues typified by structure **16** [33] possessed a favorable combination of groups, resulting in a dual AChE/BACE-1 inhibitor potentially endowed with chelating ability, thanks to the amidic portions. As a matter of fact, compound **16** was a low nanomolar inhibitor of the two target enzymes (IC_50_, AChE = 4.11 nM, BACE − 1 = 18.3 nM). No cytotoxic effect was detected in SH-SY5Y cells up to 50 *μ*M, while the balanced lipophilicity, coupled with high membrane permeability, allowed predicting a good brain penetration and metabolic stability. Moreover, the title compound was able to chelate Cu^2+^, thus having an impact on the concentration of these metal ions and their promoted neurodegeneration.

Structurally related to Donepezil on the benzylpiperidine side, compounds with general structure **17** (Figure 5) represent another recent example of MTDL, where the properties of the parent compound on AChE have been retained and then extended to BACE-1 with the introduction of properly decorated aryl groups, linked via aminic- or iminic bonds [34]. When this aryl group is represented by a 4-CF_3_ substituted ring, both the amine and the imine resulted in submicromolar inhibitors of the human enzyme isoforms and were selected for further characterization. The 4-CF_3_ substituent added higher potential to permeate through membranes, as confirmed with the PAMPA-BBB assay. The amine-based compound had also a significant effect in displacing propridium iodide from PAS-AChE, hence being more interesting for further progression. Probably due to its capacity to bind PAS-AChE, 5-20 *μ*M concentrations of this compound had antiaggregation properties not only on self-induced A*β* aggregation but also on the AChE-induced one (50% and 89%, respectively). AFM studies confirmed the reduction of A*β* aggregates after the incubation with this agent. No neurotoxic effect was observed in concentrations of up to 80 *μ*M in SH-SY5Y cells, and the effects on cognition were tested in a scopolamine-induced amnesia animal model at the maximum dose of 10 mg/kg. Both the elevated plus maze and Y-maze experiments confirmed the potential for this compound to improve spatial and immediate memory, thus having an impact on cognitive impairment. *Ex vivo* analysis evidenced attenuated levels of malondialdehyde (MDA) and increased levels of superoxide dismutase (SOD) in compound-treated animals compared to the scopolamine-treated group, suggesting antioxidant properties. A robust improvement in cognitive and memory function was also observed when the compound was evaluated in the Morris water maze experiment with an A*β*_1-42_-induced ICV rat model.

These dual AChE/BACE-1 inhibitors confirmed once again the importance of these enzymes in the pathology of AD and how the combined action against them still represent a valuable approach to address cognitive impairment and A*β*-related dysfunctions.

### 2.2. Dual ChE and GSK-3*β* Inhibitors

Glycogen synthase kinase-3*β* (GSK-3*β*) is a multitasking serine/threonine kinase largely expressed in the CNS. It is involved in several cellular processes and signaling pathways and its dysregulation occurs in the development of different disorders [35]. GSK-3*β* is also related to the pTAU phosphorylation process [36], and an increase in its activity correlates with A*β* production by interfering with APP-cleaving enzymes [37], leading to neuronal toxicity. Moreover, the overexpression of GSK-3*β* in transgenic mice is responsible for the development of cognitive deficits, thus making it a validated target in AD pathology [38]. Over the past decade, GSK-3*β* has been intensively targeted and its concomitant inhibition with AChE represents a well-consolidated and efficient approach to address the multifactorial nature of AD, influencing plaque deposition and pTAU hyperphosphorylation.

**Figure 6:**
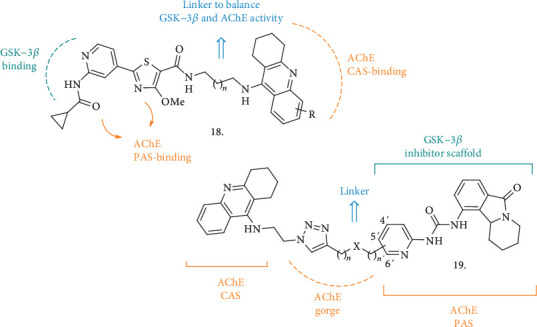
Dual AChE/GSK-3*β* inhibitors **18** and **19** and their relative pharmacophoric elements for the interaction with key enzyme residues.

From the combination of a known GSK-3*β* inhibitor [39] and the THA moiety as AChE binder, some thiazole-based compounds were synthesized (**18**, Figure 6) with the potential to be a novel class of dual GSK-3*β*/AChE [40]. An amidic bond served to link the two pharmacophoric elements, spaced by a 2- or 3-C chain, and a few substitutions on the THA aromatic ring were also assessed. The introduction of the THA moiety did not affect the potency of GSK-3*β* inhibition, and the new compounds displayed nanomolar activity against this latter enzyme and *h*AChE, with almost all of them being also selective over *h*BuChE. However, the series showed remarkable antiproliferative effects on SH-SY5Y cells and only the analogue with the unsubstituted THA moiety and a 3C-linker progressed, having an IC_50_ of 30 *μ*M against the neuroblastoma cell line and being safe in hepatocytes, with low impact on these latter cells' viability at the same concentration. This compound also showed a moderate activity against A*β* self-oligomerization. It was then tested in mouse neuroblastoma N2a-TAU cells at increasing concentrations to assess its effect on pTAU hyperphosphorylation, where it displayed a significant inhibition of this process. In the animal model of cognition impairment induced by scopolamine, the compound-treated mice displayed a significant ameliorated memory performance in the Morris water maze test, confirming the compound *in vivo* activity.

Another new series of compounds as dual inhibitors of ChEs and GSK-3*β* (**19**, Figure 6) was recently reported [41]. Here, the structure of THA (the ChE inhibitor side) and the scaffold of Valmerin (isoindolone, GSK-3*β* inhibitor side) were hybridized. The analysis of the crystal structures of the new MTDLs in complex with *Tc*AChE, combined with molecular docking studies, allowed the identification of the 1,2,3-triazole group as the best linker to retain or increase the inhibitory potency amongst both of the enzymatic systems. Together with the final hybrid compounds, the THA- and isoindolone-based fragments have also been tested for their ability to inhibit the enzymes, to give further information on the contribution of the single parts on each enzyme inhibition. The best performing compounds were able to inhibit both human AChE and GSK-3*α*/*β* in the nanomolar range, and the triazole ring undoubtedly played a pivotal role in enhancing the inhibitory potency towards the GSKs, even though the compounds were not displaying high selectivity over other kinases. Interestingly, the new analogues were less cytotoxic than the corresponding THA and isoindolone fragments in several cell lines (including the liver HuH7 cell line); however, when tested at concentrations of up to 100 *μ*M in SH-SY5Y cells, a reduction in cell viability was observed after 24 h. The viability of MDCK-MDR1 cells was not affected in the same way, so the MDCK-MDR1 cell line expressing P-gp was used as a BBB model to predict brain penetration. The new compounds displayed good permeability through this system and showed no interaction with P-gp.

All the *in vitro* and *in vivo* biological properties shown by these classes of compounds highlight a very interesting potential for the treatment of AD. Even if there is still the need of improving selectivity and lowering cytotoxicity, these hybrid structures are once again proving how different pharmacophoric elements, joined by an appropriate and specifically designed linker, represent a valuable starting point that deserves to be progressed as MTDL drugs.

### 2.3. Dual ChE and MAO Inhibitors

Another combination for a multitarget purpose arises from the dual inhibition of ChEs and Monoamino Oxidases (MAOs). In the CNS, MAOs terminate the action of several monoamine neurotransmitters, such as dopamine and serotonin, and MAO-B, the predominant isoform in the human brain, is already a validated target for neurodegenerative diseases, with its inhibitor Rasagiline being approved to treat PD symptoms [42]. The expression of MAO-B is also increased in AD patients, where a correlation between its activity and intracellular A*β* levels has been observed, possibly due to interactions with *γ*-secretase [43]. Although the role of MAO-B in AD pathogenesis remains unclear, its inhibitors have shown neuroprotective effects, thus making this enzyme an appealing target in AD [44].

**Figure 7:**
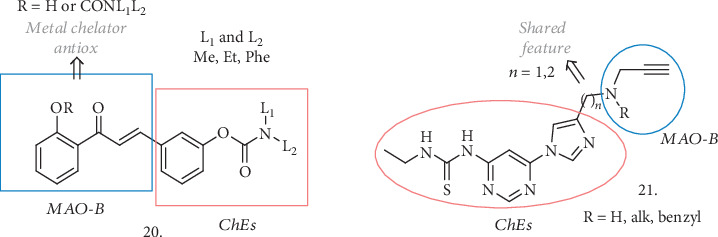
Novel ChE/MAO-B dual inhibitors and relative pharmacophoric elements. The new analogues are also endowed with structural elements which confer the ability to chelate metals and to protect against oxidative stress.

Sang et al. reported a series of chalcone-*O*-carbamate derivatives (**20**, Figure 7) potentially able to behave as ChEs and MAO-A/MAO-B inhibitors and endowed with antioxidant activities, anti A*β*_42_ aggregation and metal-chelating properties, and neuroprotective effects against H_2_O_2_-induced PC12 cell injury [45]. The new compounds are designed to combine the interesting biological activities of chalcones [46] with the well-known AChE and BuChE inhibitory activity of Rivastigmine. The addition of a hydroxyl group adjacent to the chalcone carbonyl group confers the potential to be metal-chelating agents. The combination of L1=Me and L2=Et with R=H or N(Me)Et as substituents gave the best results in terms of expected properties. The compounds were selective BuChE and MAO-B inhibitors, active in the *μ*M range for both of the enzymes, and they could inhibit the self-induced aggregation of A*β*_42_ with values higher than 50% (63.9% for the most active). A potent antioxidant activity in the Oxygen Radicals Absorbance Capacity by Fluorescence (ORAC-FL) method was observed, with the compound bearing the hydroxyl group (R=H) being more potent than its carbamate counterpart. The same hydroxyl analogue was also a selective metal chelator that could chelate Cu^2+^ and Al^3+^. Thus, its capability on Cu^2+^-induced A*β*_42_ aggregation was evaluated, displaying higher inhibition values, higher than curcumin as a reference. The two compounds were further progressed to assess their neuroprotective potential against H_2_O_2_-induced PC12 cell injury using MTT assays, where they were demonstrated to increase cell viability in correlation with their ability to capture hydroxyl radicals. Being also permeable through artificial membranes in the PAMPA-BBB assay, the compounds were finally tested *in vivo* in the scopolamine-induced cognitive impairment assay. The hydroxyl-derivative was effective in improving short-term working memory in mice, even though at the highest dose (23.4 mg/kg) it showed some neurotoxic effect.

Xu et al. have also presented a nice example of propargylamine-modified scaffolds (**21**, Figure 7) as ChE and MAO inhibitors [47]. In more detail, they combined the imidazole-substituted pyrimidinylthiourea moiety (AChE inhibitor pharmacophore) with the propargylamine group of Selegiline (MAO-B inhibitor pharmacophore), spaced by different linkers. All the compounds resulted in submicromolar inhibitors of AChE with negligible activity on BuChE, and the R=H or Me substitutions were the most appropriate for an efficient inhibition, especially when coupled with a single carbon atom linker (*n* = 1). Following these encouraging preliminary results, MAO inhibition was tested, revealing that the abovementioned compounds efficiently inhibited the enzymes in the micromolar range, and with the R=Me compound being selective for MAO-B. It also showed good antioxidant activity in the ORAC-FL assay, and the thiourea fragment worked as the metal-chelating part, resulting in a selective chelation for Cu^2+^ and inhibition of the ROS produced by Cu(II)-related redox. This compound had no effect on the A*β* self-aggregation but could efficiently inhibit Cu-mediated A*β* aggregation, as expected. It was safely tolerated on rat primary cortical neurons at concentrations of up to 30 *μ*M, showing mild neurotoxicity at 100 *μ*M, and it could protect neuronal cells from Cu-induced A*β* toxic damage, increasing cell viability. The PAMPA assay indicated a good potential to cross the BBB, and the *in vivo* effect in the scopolamine-induced cognitive deficit in mice was evaluated. The HCl salt was dosed orally at 30 mg/kg, and it could ameliorated learning and memory deficits, with the treated mice showing shorter escape latency and less frequent errors compared to the scopolamine group.

These data demonstrated a promising profile for these dual ChE/MAO inhibitors, and together with other described series of compounds, they are worth of further development and analysis in additional analysis in animal models of dementia associated to neurodegenerative conditions.

### 2.4. ChE Inhibitors and Other Enzymatic Systems

**Figure 8:**
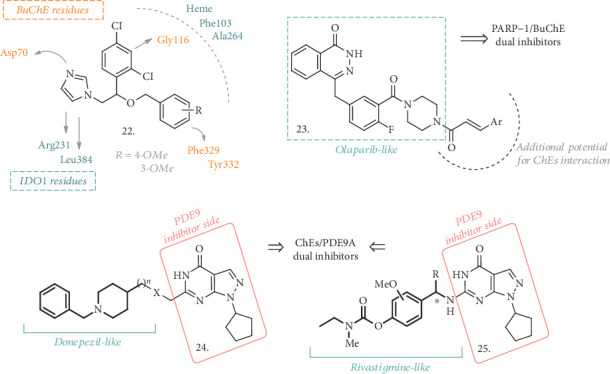
Structures of dual BuChE/IDO1, ChE/PARP-1, and ChE/PDE inhibitors. While structure **22** is inspired by Miconazole, Olaparib has been taken as the reference structure in the synthesis of MTDL **23**, while Donepezil and Rivastigmine, coupled with the pyrazolopyrimidinone structure of a known PDE9A inhibitor led to the identification of compounds with general structures **24** and **25**.

The simultaneous inhibition of ChEs and indoleamine 2,3-dioxygenase 1 (IDO1) resulted in another target combination endowed with beneficial effects in AD. IDO is an intracellular cytosolic heme-containing enzyme that regulates the degradation of Tryptophan (Trp) to N-formylkynurenine in the kynurenine pathway (KP), acting as a first-step rate controller [48]. KP is unbalanced in some neurodegenerative disorders and, as a result, Trp catabolism leads to neurotoxic metabolites such as 3-hydroxykunurenine. IDO1 is essential for this pathway, and its activation has been linked with A*β*-related inflammation in AD [49], making it the focus of various researches on neurodegenerative diseases treatment [50–51]. Lu et al. have identified a novel structure endowed with double activity on BuChE and IDO1 [52]. The selective activity on BuChE is of particular interest, as its levels rise up in the advanced stages of AD, replacing AChE deficiency in the hydrolysis of Ch, thus becoming an even more important target. Compounds with general structure **22** (Figure 8) are prototypes based on the antifungal drug Miconazole, which had already shown activity as an IDO1 inhibitor [53] and was tested in vivo as a reference compound. The 3-OMe or 4-OMe-substituted analogues showed the weakest activity on AChE and the best inhibitory IC_50_ values on BuChE and IDO1 (8.3 and 2.8 *μ*M for the 3-OMe and 16.5 and 1.0 *μ*M for the 4-OMe, respectively), being more potent than Miconazole. Their effect on spatial memory was assessed in the scopolamine-induced impairment in mice, where they displayed better performances than the control group treated with THA. Moreover, no acute hepatotoxic damage was observed. Taken together, all these characteristics highlight the favorable potential for these structures to combine interesting activities on innovative AD-related targets.

Another interesting approach in the search for MTDLs comes from Gao et al., who presented dual ChE and Poly(ADP-ribose) Polymerase-1 (PARP-1) inhibitors [54]. PARP-1 inhibitors have been extensively studied for their anticancer activity [55], but they may also serve as potential therapeutics for neurodegenerative diseases, with particular attention to AD and PD [56]. The role of PARP-1 has not yet been fully elucidated, but there are emerging evidences for the neuroprotective effect of its inhibitors. To merge the PARP-1 inhibition with that on ChEs, the structure of the known PARP-1 inhibitor Olaparib has been selected and modified with the introduction of substituted aryl vinyl groups in the place of the cyclopropane one (**23**, Figure 8). This combination should guarantee the dual activity on PARP-1 and ChEs, since it led to the formation of 3-aromatic-*α*,*β*-unsaturated carbonyl moieties, which are recurring groups in several natural ChE inhibitors and bioactive/neuroprotective compounds. The *in vitro* test revealed that all the analogues of the series were low micromolar inhibitors of PARP-1, even though none of them was more potent than the parent compound Olaparib. However, they showed moderate micromolar activity against AChE and BuChE, being more potent inhibitors of this latter enzyme. When Ar was a 3- or 4-nitrosubstituted phenyl ring, the highest inhibitory potency against BuChE was achieved, with an IC_50_ of 9.2 and 5.9 *μ*M, respectively, even greater than Neostigmine as the reference. The abovementioned analysis confirmed the dual activity of these analogues and, although they may not be potent enough for *in vivo* analysis, molecular docking studies have already helped find a way to improve their activity. Thus, another interesting class of MTDLs may arise from the combination of these pharmacophoric elements leading to dual ChE and PARP-1 inhibition.

Phosphodiesterases have recently gained interest for their potential to ameliorate cognitive functions in AD patients. These enzymes can be involved in the regulation of cAMP and cGMP levels [57], and their inhibition can offer a valuable tool to increase the levels of the two second messengers in the hippocampus and cerebral cortex, with a consequent improvement in the memory and learning processes. Phosphodiesterase-9 (PDE9), which exerts its hydrolytic activity against cGMP, is now being studied as a potential target for CNS disorders [58], including AD, and the recently developed inhibitor PF-04447943 [59] has been tested in Phase II clinical trials for AD treatment (NCT00930059).

To assess the effects of dual PDE9A/AChE inhibitors, Hu et al. [60] exploited the benzylpiperidine moiety of Donepezil in combination with the pyrazolopyrimidinone structure (**24**, Figure 8) of a reported PDE9A inhibitor [61]. Although different amidic or (cyclic)amine chains were explored as linkers for the two pharmacophores, the best results were obtained with 4-member ethereal or carbon tethers, resulting in compounds with submicromolar inhibitory activity against PDE9A and AChE. The type and length of the linkage played a pivotal role in the inhibition of PDE9A and was also responsible for the mixed type of inhibition of AChE, allowing the binding to both catalytic anionic site (CAS) and peripheral anionic site (PAS) of the enzyme. No effect was observed on SH-SY5Y cell viability at concentrations between 10 and 20 *μ*M, demonstrating a limited neurotoxic potential. All the compounds were tested in the PAMPA assay, where the results showed that they were also potentially able to cross the BBB. Despite the moderate metabolic stability, acute toxicity was evaluated and, following the lack of detrimental adverse events, the compounds were tested *in vivo* in the scopolamine-induced mice model of cognitive and learning deficits. These analogues could significantly improve spatial memory and cognition in the Morris water maze tests; thus, they were also investigated in a mice model of spatial learning and memory deficits produced by an ICV injection of A*β*_25−35_. Once again, there was a partial amelioration of the deficits induced by the treatment with the best performing compounds of the series.

Following a similar approach, hybridization of the pyrazolopyrimidinone skeleton with Rivastigmine led to the identification of another series of promising MTDLs (**25**, Figure 8) [62]. Even if various groups were used to replace the carbamate functionality of Rivastigmine and different chains were employed to create the linkage between the two pharmacophoric elements, compounds with general structure **25** resulted as the most promising and efficacious analogues of the series. Interestingly, these agents were behaving as selective inhibitors of BuChE, with IC_50_ values ranging from 0.96 to 18.8 *μ*M, and were also potent PDE9A inhibitors in the nanomolar range. A little study on the selectivity over the PDE superfamily was also reported, confirming a good degree of selectivity for the PDE9A enzyme. Nevertheless, carbamate compounds were not active as antioxidants, and only the replacement of this functionality with hydroxyl groups restored the antioxidant potential. In addition, some of the tested compounds were not cytotoxic in SH-SY5Y cells in concentrations of up to 100 *μ*M and were able to inhibit A*β* self-aggregation to some extent at a concentration of 50 *μ*M.

All these data pointed out that the combination of the pyrazolopyrimidinone with other pharmacophores of ChE inhibitors is an optimal strategy to develop novel candidates for the treatment of AD.

### 2.5. ChE Inhibitors and NMDA Receptor Antagonists or Ca^2+^ Channel Blockers

The simultaneous inhibition of ChEs and the antagonistic effect on *N*-methyl-*D*-aspartate receptors (NMDARs) is definitely one of the most promising strategies towards the identification of new MTDLs. NMDA ionotropic receptors are activated by the excitatory neurotransmitter glutamate, and they are permeable to different positive ions, including Ca^2+^, thus contributing not only to synaptic plasticity and long-term changes but also to the excitotoxicity process [63]. When the concentration of intracellular Ca^2+^ reaches pathological levels, there is a loss of synaptic functions and neuronal cell death, with a progressive cognitive decline. Recently, the activation of NMDARs has been linked to AD-related synaptic dysfunctions [64] and the NMDAR noncompetitive antagonist Memantine has been approved as symptomatologic treatment for moderate to severe AD [65]. The combination of NMDAR antagonism with the inhibitory activity on ChEs may have beneficial or synergic effects on AD symptomatology, as already proven in animal models of AD [66, 67]. Moreover, a fixed-dose combination of Donepezil and Memantine (known as the drug Namzaric®) is now used to treat moderate to severe dementia stages associated with AD [68].

**Figure 9:**
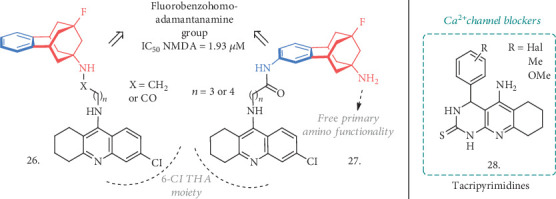
The novel THA-adamantanamines **26** and **27** acting as ChE inhibitors and NMDAR antagonists. Also, tacripyrimidines with general structure **28** are here represented as agents influencing Ca^2+^ influx and intracellular concentration by blocking voltage-gated Ca^2+^ channels.

In line with this hypothesis, a new series of benzohomoadamantane-chlorotacrine hybrids has been proposed as novel ChE inhibitors and brain penetrant antagonists for NMDARs. Following their previous knowledge in the development of THA-based AChE and BuChE inhibitors and polycyclic amines as antagonists of NMDA receptors, the authors exploited different linkers, varying their lengths and linkage positions, to connect the benzohomoadamantane motif with a 6-chlorotacrine (**26** and **27**, Figure 9) [69]. In particular, the two linkage positions were represented by the bridgehead amino group on the benzene ring of the benzohomoadamantane core (**26**) or by an additional amino group on the same system (**27**). This resulted in the identification of novel MTDLs, potentially able not only to inhibit ChEs and to antagonize NMDA receptors but also to interfere with BACE-1 activity and A*β*_42_ and pTAU aggregation, as already reported for the two separate pharmacophoric elements.

In both of the series, the new compounds retained the activities of the parent analogues on the primary targets, being AChE and BuChE inhibitors in the subnanomolar and submicromolar ranges, respectively, and binding to NMDA receptors in the micromolar range. Nevertheless, there was no activity on other proteins or targets associated with AD, as it was originally expected for the association of the two moieties. However, the increased potencies of some agents compared to the reference compounds represent a valid reason for a more in-depth evaluation in the anti-AD drug discovery field.

Another remarkable step forward for the discovery of new MTDLs took into account the possibility of creating structures that could merge the ChE inhibitory activity with the calcium channel blockade ability, thus limiting the entrance of Ca^2+^ through voltage-gated channels (VGC) and preventing neuronal damage [70]. To this aim, a series of tacripyrimidines have been proposed (**28**, Figure 9) [71], whose structures arise from the hybridization of THA with 3,4-dihydropyrimidin-2(1H)-thiones, known to be efficient calcium channel blockers. The 3-Br substituted analogue resulted as the most potent and selective *h*AChE inhibitor with IC_50_ = 0.037 *μ*M, although the 3-methoxyphenyl derivative also showed good *μ*M activity. The selectivity over BuChE was generally high, with the 4-(halo)-substituted compounds being slightly more potent against this enzyme. The 4-Cl derivative showed a reverted trend and was more potent on BuChE, while the 3-OMe-substituted compound was equipotent on the two enzymes. Further analysis revealed a noncompetitive type of inhibition, and molecular docking studies helped establish the key interactions of these compounds in the enzyme's CAS and PAS, pointing out the halogens as important substitutions for the activity and selectivity over the ChEs. The Ca^2+^-channel blockade was investigated in SH-SY5Y cells by measuring the Ca^2+^ influx induced by K^+^ depolarization. All the tacripyrimidines inhibited Ca^2+^ influx, with the most promising and potent inhibitors of AChE showing blockade activity similar to the reference Nomodipine. The compounds were relatively safe on HepG2 cells up to 100 *μ*M, even if some derivatives showed higher hepatotoxicity than THA at higher concentrations. There was no significant effect on self-induced A*β* aggregation, as expected, but predicted ADMET properties showed a favorable potential for this candidate to be evaluated *in vivo*. The best performing tacripyrimidine of the series had a balanced activity over the selected targets and good ADME properties coupled with a lower toxicity than THA, thus making it an attractive structure deserving further investigation and development as an MTDL in AD.

### 2.6. ChE Inhibitors and Serotonin Receptor Modulators (5-HT_4_ and 5-HT_6_)

Serotonin and its receptors (5-HTRs) have been conferred notable attention during the past decades, especially for their peculiar distribution in brain areas connected to memory and learning and thus for the role played by this system in cognition [72]. Hence, the modulation of specific 5-HTR subtypes could represent a major therapeutic strategy in the fight against AD [73]. Selective ligands for 5-HT_4_R and 5-HT_6_R have been the principal focus of the latest researches, and the effects of 5-HT_4_R agonists and 5-HT_6_R antagonists (or a combination of these efficacies) have been evaluated [74].

5-HT_4_Rs belong to the G-protein-coupled receptor family and are localized both in peripheral areas and in the CNS, with a high density in the substantia nigra, striatum, and hippocampus. Here, they act as modulators of hippocampal synaptic responsiveness and plasticity, thus playing a central role in information storage and cognition [75]. Therefore, 5-HT_4_R agonists have the potential to be therapeutically useful in AD not only for the effects on memory and behavioral performances, but also because 5-HT_4_Rs influence the cholinergic system and ACh release, and are linked to APP activity and A*β* production [76]. 5-HT_6_Rs are also G-protein-coupled receptors whose expression is restricted to the CNS. Although the information regarding their pharmacology is still limited, the presence of 5-HT_6_R in brain areas responsible for cognitive functions, memory, and learning (such as hippocampus and cerebral cortex) made them another interesting target in AD [77]. Antagonists of these receptors become attractive therapeutics to address some of the AD-related dysfunctions [78], also in correlation with their procholinergic effect which is enhanced by the combination with approved AChE inhibitors.

**Figure 10:**
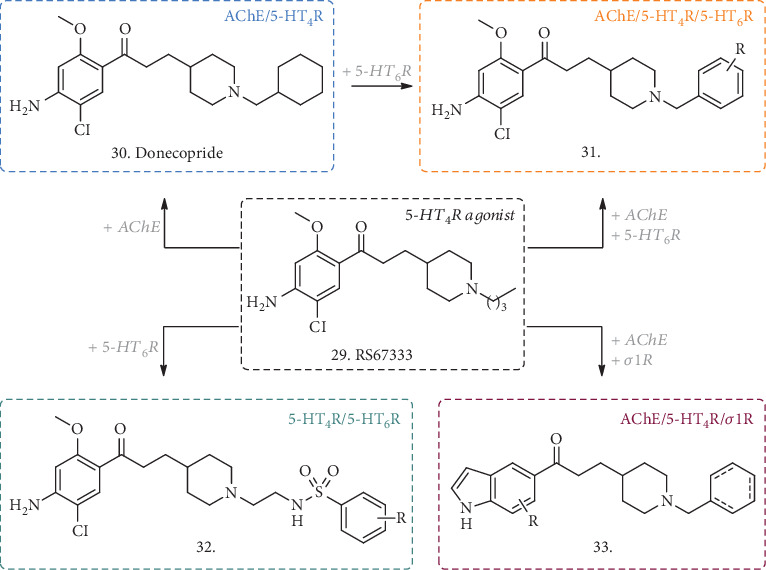
The discovery of Donecopride (**30**) inspired by the 5-HT_4_R agonist RS67333 (**29**) and the structurally related MTDLs acting as ChE inhibitors and/or serotonin 5-HT_4_ and 5-HT_6_ receptor modulators (**31** and **32**). The general structure **33** has been added as a further modification of the scaffold of RS67333 and Donecopride, although its activity shifted from 5-HTRs to *σ*1R.

With the aim of merging the anti-ChE activity and the modulation of 5-HT_4_/5-HT_6_ receptors, different structural combinations have been explored. Recently, the same group that discovered Donecopride (**30**, Figure 10) was able to develop a novel class of MTDL able to inhibit AChE, activate 5-HT_4_R, and block 5-HT_6_R. Donecopride [79] is an AChE inhibitor with partial 5-HT_4_R agonist activity inspired by the 5-HT_4_R agonist RS67333 (**29**, Figure 10), which showed *in vivo* procognitive and antiamnesic effects in NMRI mice and promoted sAPP*α* release in C57BL/6 mice [80]. Through minor structural modifications of the benzyl analogue of Donecopride, a few derivatives were obtained with very promising *in vitro* triple effects (**31**, Figure 10) [81] with R=3-Me substitution (as a fumarate salt), the compound possessed an interesting profile with *K*i_(5 − HT4R)_ = 210 nM and *K*i_(5 − HT6R)_ = 219 nM and IC_50_ on AChE = 33.7 nM, acting as a partial agonist towards *h*5-HT_4_R (similar to Donecopride) and as an inverse agonist towards *h*5-HT_6_R. Moreover, *in vivo* evaluation on an NMRI mice model demonstrated an antiamnesic effect at a dose of 0.3 mg/kg, with no detrimental effects at concentrations higher than 100 mg/kg, thus representing a quite promising MTDL candidate for AD treatment.

Further structural modifications of Donecopride allowed the discovery of other interesting compounds endowed with a multitarget profile, such as a dual 5-HT_4_R partial agonist and 5-HT_6_R antagonist, with no activity on AChE (**32**, Figure 10) [82] useful to study the serotoninergic role in AD, or a dual inhibitor of AChE and modulator of *σ*1 receptor (*σ*1R) (**33**, Figure 10), that will be subject of discussion in the following paragraph as a more recent combination to address AD-related dysfunctions.

**Figure 11:**
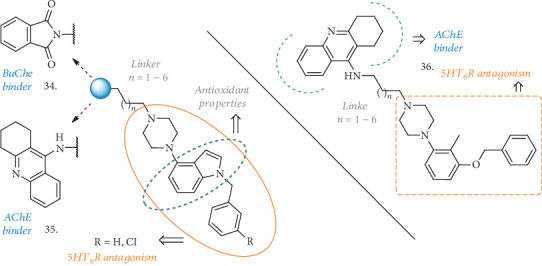
General structures of recent ChE inhibitors/5-HT_6_R antagonists. The pharmacophoric elements for the dual activity are highlighted for each structure. Together with the multiple activity, some of these agents are also endowed with antioxidant activity and effects on A*β* aggregation.

Another series of compounds reported by Marcinkowska et al. merged an *N*-benzylindole-piperazine skeleton with phthalimide- [83] or THA-moieties [84] (**34** and **35**, Figure 11), using carbon tethers with different lengths as linkers. While the indole-piperazine section brought the potential antagonism for 5-HT_6_R [85], indole alone may have antioxidant properties and the phthalimide or THA groups could contribute to ChEs inhibition, thus creating hybrid compounds with a multifaceted activity. In the phthalimide subseries (**34**), the new compounds displayed affinity for 5-HT_6_R with *K*i ranging from 21 to 252 nM and a clear correlation of the binding potency with the length of the linkers with phthalimide. Cell functional studies confirmed the antagonistic activity of these derivatives. Moreover, they were micromolar inhibitors of BuChE, with no or slight activity on AChE, and once again shorter linkers performed better than the longer ones, with R=H unsubstituted analogues being slightly more potent than the halogenated counterparts. The antioxidant activity was determined by FRAP assay, where all the compounds showed antioxidant potential starting from 10 *μ*M, and some of them had even better activity than the reference ascorbic acid.

When the THA moiety (**35**) was in the place of the phthalimide one, the R=H compounds showed a higher nanomolar activity against AChE and, to the same extent, on BuChE, becoming nonselective and noncompetitive ChE inhibitors, but still deserving further development for AD treatment. The antagonist activity on 5-HT_6_R was also conserved, and the 5-C atom chain gave the best *K*i value of 72 nM. The thioflavin-T (ThT) assay on A*β* self-aggregation revealed equal or better inhibitory potency (over 92%) than resveratrol used as a reference, and PAMPA-BBB prediction determined a favorable potential to diffuse across the membranes.

Another combination of the THA moiety with a tolylamino fragment, known to be a 5-HT_6_R antagonist [86], and spaced by the same linkers (**36**, Figure 11) displayed similar activities on the abovementioned systems [77]. In this case, *K*i values on AChE and BuChE were even higher in comparison with the previous structures (reaching 10 nM and 22 nM for the best analogue, respectively) and still showing potent binding and antagonism towards 5-HT_6_R. Together with the positive effects on A*β* self-aggregation and a good predicted permeability across membranes, the *in vitro* metabolic stability of these analogues in human liver microsomes highlighted a of 120 min with none of the known THA-related hepatotoxic metabolites identified.

Even if these compounds have not been tested *in vivo* in animal models of cognitive disorders, their *in vitro* analysis revealed a high potential to be useful MTDLs deserving further evaluation in AD.

### 2.7. ChE Inhibitors and H_3_R Antagonists or *σ*1R Agonists

Besides the more classical couplings of ChE inhibitors with the previously described receptor families, other appealing combinations have been recently explored. So far, the histamine H3 receptor (H_3_R) has been the focus of numerous researches for the treatment of cognitive disorders [87]. H_3_R are mainly expressed in the cortex, hippocampus, caudate, and putamen [88], and their activation influences the release of different neurotransmitters (including AChE), thus having an impact on brain disorders like AD. For this reason, antagonists of H_3_R have been investigated for their potential role in cognitive dysfunctions related to AD [89]. As mentioned above, *σ*1Rs also found their place in the context of neurodegenerative disorders [90]. These receptors are mainly situated in the endoplasmic reticulum where they normally exert prosurvival and antiapoptotic effects, but they can be found in other organelles influencing lipid, protein, and ion trafficking [91]. Changes in the function or expression of this multifunctional protein have been linked to various diseases, including AD [92] and HD. Here is why some examples of ChE inhibitors in combination with H_3_R antagonist or *σ*1R agonist activities have been proposed.

**Figure 12:**
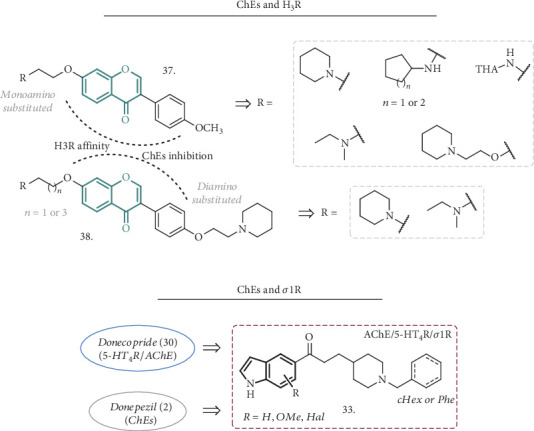
General structures of novel combinations for the discovery of dual ChE inhibitors with H_3_R antagonist (**37** and **38**) or *σ*1R agonist (**33**) activities.

Wang et al. presented some novel isoflavone derivatives bearing amino or THA groups linked to the 7-position of the isoflavone core by different chains, along with some diamino-substituted analogues inspired by known H_3_R antagonists (**37** and **38**, Figure 12) [93]. A preliminary analysis of the H_3_R antagonist profile made on isoflavone-based compounds with known AChE inhibitory activity guided the identification of the appropriate linkers and amines for SAR studies. Even if the monoamino substituted derivatives (**37**) showed good activities on the selected targets, the analogues bearing a second amino functionality (**38**) resulted as the more potent low micromolar antagonists of H_3_R. They also displayed pronounced dual AChE and BuChE inhibition at the low- or submicromolar level, with the best inhibitor having IC_50_ values of 0.08 *μ*M and 2.9 *μ*M, respectively. These compounds also showed antioxidant activity in the ORAC-FL test and possessed an anti-inflammatory effect in LPS-stimulated BV-2 microglia cells, suppressing the production of IL-6 and TNF-*α* without affecting cell viability. Moreover, they were not cytotoxic in SH-SY5Y cells in concentrations of up to 100 *μ*M, and in the SH-SY5Y-APPsw cell line (overexpressing the Swedish mutant form of human APP), they significantly prevented copper-induced A*β* aggregate toxicity, increasing cell viability. Acute toxicity was assessed in mice, resulting in the toleration of up to 1000 mg/kg, and the favorable PK parameters and drug-like properties prompted the authors to test the compounds in animal models of cognitive deficits. In mice, there was a significant prolongation of the scopolamine-induced latency of step-down in a dose-dependent manner (10 to 30 mg/kg) and an increase in brain cholinergic activity, ameliorating cognitive deficit. All together, these results highlighted an exquisite multitarget profile for these compounds and the *in vivo* analysis suggested an interesting potential for the treatment of AD.

Starting from the structure of the previously described Donecopride (**30**, Figure 10) [79], Lalut et al. focused their research on novel ligands endowed with AChE inhibitory activity and *σ*1R affinity [94]. Modifications to the aromatic region of the reference compound with the introduction of a substituted indole ring (**33**, Figure 12) afforded a different receptor affinity profile, where the activity on 5-HT_4_R was partially lost while the interaction with *σ*1R become the pivotal feature for the new analogues. Together with the binding affinity for *σ*1R, the indole scaffold was also increasing the interactions with the peripheral anionic site (PAS) of AChE.

All the compounds were evaluated for their ability to inhibit *h*AChE and to bind to guinea pig (gp) 5-HT_4_R. They showed an overall decrease in 5-HT_4_R affinity, with the *N*-benzylpiperidines being the weakest ones, although some analogues changed their functional activity on the receptor, acting as an antagonist instead of a partial agonist, as Donecopride was. They all displayed a potent inhibition of AChE, with the *N*-benzyl groups on piperidines enhancing this activity, behaving as noncompetitive inhibitors, suggesting a possible interaction with the PAS and the subanionic site of the active site. These interactions were subsequently confirmed by the X-ray analysis of the crystal structures of selected compounds with the enzyme. Regarding the *σ*1R affinity, most of the compounds of the series were very potent ligands of this receptor, and the presence of the indole ring was crucial for the activity. One of the best compounds (IC_50_ (AChE) = 28.8 nM, *K*i (5 − HT_4_R) = 37.5 nM, and *K*i (*σ*1R) = 5.1 nM) was also evaluated *in vivo* in a dizocilpine-induced amnesia model, showing a protecting effect in the passive avoidance test that correlates with its *in vitro* potent *σ*1R affinity. For all these reasons, this new series of compounds has a great potential to be useful in the treatment of AD and deserves further investigation.

### 2.8. The Effects of Dual ChE Inhibitors and Modulators of the Endocannabinoid System

Another innovative approach in the search for MTDLs envisages the possibility to act on ChEs or BACE-1 and to couple this activity with the modulation of the endocannabinoid system (ECS). The ECS is composed of endogenous lipid-signaling molecules defined as endocannabinoids (ECBs) and their cellular targets, the G-protein-coupled cannabinoid receptors (type-1 and type-2 CBRs), along with the transporters and enzymes responsible for ECB biosynthesis and metabolism. *N*-Arachidonoylethanolamine (anandamide (AEA)) and 2-arachidonoylglycerol (2-AG) are two members of the ECB signaling molecules, and they activate CBRs to modulate a wide range of responses and processes including pain, inflammation, and thermoregulation [95]. The actions of these signaling lipids are rapidly terminated by cellular reuptake and subsequent hydrolysis operated by a number of enzymes. Amongst the latter, the Fatty Acid Amide Hydrolase (FAAH) was originally identified as the enzyme responsible for AEA hydrolysis [96] while the Monoacylglycerol Lipase (MGL) plays a pivotal role in the regulation of 2-AG levels [97]. ECB signaling has been found altered in some neurodegenerative diseases. Evidences pointed out to how decreased levels of AEA, for example, correlate with an inverse trend to those of A*β* [98]. In addition, CB2R, which is associated with immune system and microglia activation during neuroinflammation, is selectively expressed in areas of neuritic plaques, suggesting a potential role for this receptor in the inflammation associated with AD [99]. These findings suggested that the modulation of ECS may have a profound impact on AD.

**Figure 13:**
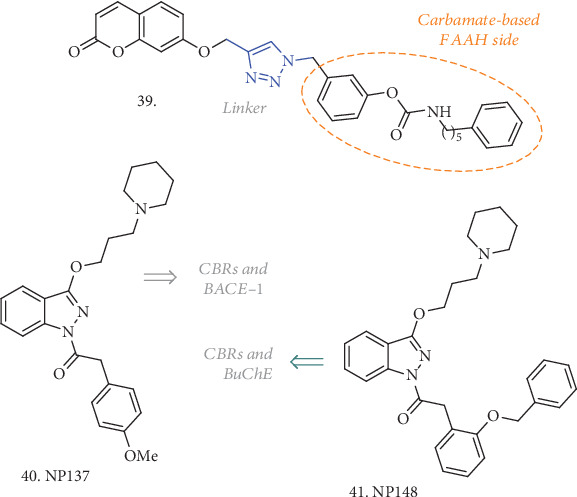
Structures of the novel compounds acting on ChEs (and BACE-1) and able to modulate the ECS via direct interaction with CBRs (**40** and **41**) or by increasing the ECBs' tone through the inhibition of the AEA-metabolizing enzyme FAAH.

ECS can be modulated either by direct stimulation of CBRs or by inhibition of the ECB catabolic enzymes, leading to increased levels of ECBs [100–104]. For this latter purpose, Montanari et al. have recently proposed some compounds, here represented by **39** (Figure 13), endowed with inhibitory activity towards FAAH, AChE, and BuChE [105]. They evaluated several structures where the triazole linker of **39** was replaced by a more flexible *N*-methylalkyl chain, some of which were able to inhibit both AChE and BuChE to a similar extent, and to retain a good activity towards the FAAH enzyme. However, a more balanced profile was achieved with **39**, having an IC_50_ against *r*FAAH, *h*AChE, and *h*BuChE of 922, 42.7, and 27.9 nM, respectively, and thus displaying good selectivity for BuChE. Cytotoxicity evaluation on SH-SY5Y cells up to 50 *μ*M showed no acute toxicity and, although no in vivo evaluation was performed, these compounds are worth further investigation for AD treatment.

With the aim of discovering MTDLs for AD treatment able to modulate ECS via direct CBR stimulation, Nuñez-Borque et al. have identified two CBR agonists which also act as BACE-1 and/or BuChE inhibitors [106]. No *K*i values were reported for CBRs, but functional experiments confirmed the agonist profile for **40** and **41** (Figure 13), and while **40** had a 60% inhibition of BACE-1 at 10 *μ*M, compound **41** had a 38% inhibition at the same concentration and an IC_50_ on BuChE of 2.5 nM. In rat primary cortical neuronal cultures, both compounds efficiently attenuated A*β*-induced cell death, increasing cell viability, while only **40** was able to improve performances in an animal model of AD, namely TgAPP transgenic mice. Moreover, this compound was able to restore abnormal features of the AD lymphoblast, thus having an impact on nonneuronal cell cycle alterations, considered systemic manifestation of the disease. This was achieved either by preventing the enhanced serum stimulation of cell proliferation or by sensitizing cells to apoptosis in conditions of higher resistance to serum deprivation-induced cell death. More detailed studies are needed to completely understand the effects and mechanisms behind these MTDLs, but they represent a nice way to address AD from a different perspective.

### 2.9. ChE Inhibitors with Multiple Effects on A*β*-Aggregation, Metal-Induced Toxicity, and Oxidative Stress

Even when not coupled with other enzymatic activities, ChE inhibitors may have a multitarget profile if considering (*i*) their possible action against A*β* aggregation, (*ii*) their disaggregation effect on preformed A*β* fibrils, and (*iii*) the metal-chelating properties, affecting metal dyshomeostasis and oxidation processes. This combination often resulted in compounds with the potential to be neuroprotective MTDLs.

**Figure 14:**
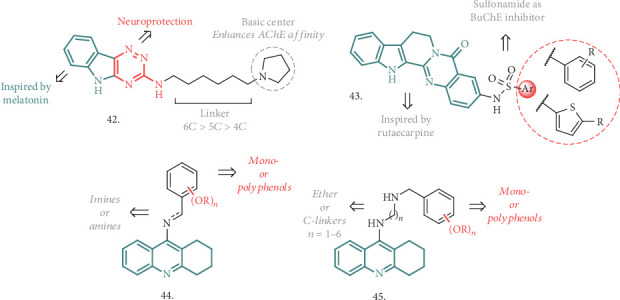
Some of the most recent compounds which combine ChE inhibition with the neuroprotective effects, acting on the classical hallmarks of AD, such as A*β* aggregation/disaggregation, metal-induced toxicity, and oxidative stress.

In the search for ChE inhibitors with additional neuroprotective and antioxidant properties, Patel et al. reproposed the exploitation of the indole ring to build up novel multiactive structures, guided by the observation that melatonin, based on the same indole moiety, is endowed with free radical scavenging ability and neuroprotection against A*β*-induced toxicity [107]. They merged this ring with the 1,2,4-triazine scaffold, which is also a common feature for several drugs, and then explored the effects of thio- and amino-linked aryl/benzyl/aminoalkyl side chains (typified by **42**, Figure 14). All the compounds showed micromolar to submicromolar activity towards AChE and BuChE. Some of the most active analogues (IC_50_ < 5 *μ*M) showed antioxidant activity in the 1,1-diphenyl-2-picrylhydrazyl (DPPH) assay at concentrations ranging from 10 to 20 *μ*M, with moderate to good free radical scavenging activity (54.9-64.3%) compared to ascorbic acid. No cytotoxicity was observed in the SH-SY5Y cell line (up to 80 *μ*M) and in H_2_O_2_- and A*β*-induced toxicity studies; compound **42** exerted a substantial protection against the toxic insult in a concentration-dependent manner. Besides a very detailed computational analysis to establish the essential interactions and features required for the multitarget activity, confirming the key role of the triazinoindole core and the enhancement brought by the introduction of a basic center in the chain, the compound was tested for its cognitive improvement effect in animal models of AD. In scopolamine-induced amnesia in rodents, it showed spatial memory improvement in the Morris water maze learning test at doses of 5 and 10 mg/kg p.o. Moreover, in the A*β*-induced AD model, the lowered spontaneous alternations induced by A*β*_1−42_ were significantly reversed at the same doses. The neurochemical analysis carried out on the scopolamine- and compound-treated animals contributed to confirm the ability of **42** to reverse the reference and working memory deficit as well to manage the oxidative stress-induced dementia. If coupled with the notable *in silico* ADMET properties, all these analyses highlighted the favorable potential for this analogue to be a useful treatment for AD-related deficiencies.

The combination of a sulphonamide moiety with the core structure of Rutaecarpine, a well-known pentacyclic indolopyridoquinazolinone alkaloid from Chinese medicine, has been proposed by Wu et al. as a promising MTDL (**43**, Figure 14) [108]. All these analogues had good to moderate concentration-dependent activity against BuChE in the *μ*M range, being more potent than the parent compound but not as efficient as THA. They also resulted to be selective inhibitors of the abovementioned enzyme, with almost a null effect on AChE. At a concentration of 100 *μ*M, there was a significant effect in the DPPH free radical scavenger assay, with ascorbic acid and Donepezil used as reference antioxidants, underlying a mild scavenging activity. There was also a robust effect against intracellular ROS generation in H_2_O_2_-treated SH-SY5Y and PC12 cells, where the compound treatment restored the ROS levels almost to the blank group, highlighting a neuroantioxidant potential. The same models and cell lines were used to assess neurotoxicity by measuring cell viability after incubation with increasing concentrations of the title compounds. In both of the cases, survival rate increased in a dose-dependent manner. Coincubation of A*β*_42_ with the best performing analogue of the series resulted into interference with the peptide self-assembly process, and TEM analysis confirmed an antiaggregation effect comparable or superior to that of Donepezil at concentrations of 100 *μ*M. The presence of the carbonyl and sulfonamide groups in the compounds may have conferred chelating properties, and differences in the UV absorption at a peculiar wavelength indicated the capability to chelate Cu^2+^ in a 1 : 1 stoichiometry. The newly introduced features in the Rutaecarpine scaffold led to a combination of interesting activities, placing this core and its analogues under the spotlight for further development as MTDLs against AD.

Another recent research work evaluated the possibility of using different tethers to combine the THA motif with (poly)phenolic or methoxy-substituted rings, generating novel MTDLs potentially useful for AD treatment (general structures **44** and **45**, Figure 14) [109]. In more detail, the new analogues were designed to overcome classical THA-derived side effects and to provide access to other significant therapeutic targets, such as neuronal redox status, deposition of amyloid plaque, leading to neuroprotection. The linkage strategy, guided by molecular docking analysis, envisaged the formation of imino-, amino- (**44**), or ethereal (**45**) bonds, coupled with the variation of the tether length by increasing the number of carbon atoms. The best performing analogue of series **44**, bearing a 9-atom ether-type chain and a dimethoxy-substituted ring, behaved as an extremely potent (subnanomolar range) and selective BuChE inhibitor, with an 85-fold increase of activity compared to THA, and displayed a good ability to interfere with A*β* self-aggregation, lacking neurotoxicity at concentrations of up to 5 *μ*M. Its neuroprotective properties were assessed in primary rat neurons, inducing neuronal damage by serum and K^+^-deprivation, where it showed neuroprotection at concentrations of up to 10 *μ*M. All these activities, combined with a low hepatotoxicity and good stability under physiological conditions, pointed out to this lead compound as a promising pharmacophore combination deserving further analysis and progression in the list of AD treatment.

### 2.10. BACE-1 Inhibitors and Their Combinations into MTDLs

As already discussed in the previous sections, BACE-1 represents another key enzyme targeted in AD and the importance and effects of dual ChE/BACE-1 inhibitors have been reported (Section 2.1). In the search for MTDLs, other interesting combinations may arise from the exploration of novel BACE-1 inhibitors endowed with neuroprotective and anti-inflammatory effects.

**Figure 15:**
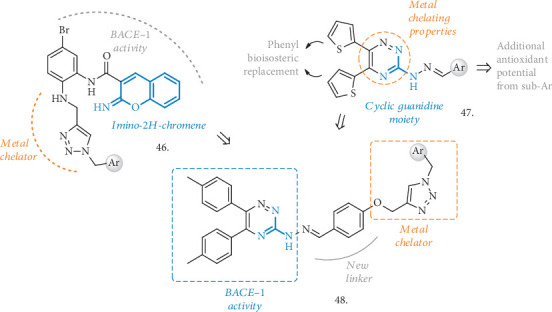
BACE-1 inhibitors with metal-chelating properties and radical scavenging potential.

Inspired by coumarine, acylguanidine, and cyclic guanidine moieties, different series of compounds have been identified as BACE1 inhibitors, also endowed with antioxidant and metal-chelating activities (**46-48**, Figure 15). Some of them arise from the combination of the phenylimino-2H-chromen core with an aminomethylene-1,2,3-triazole ring (**46**), while the use of the 3-hydrazynyl-1,2,4-triazine structure led to the identification of compounds **47** and **48**. The latter also prompted the authors to evaluate the contribution of the 1,2,4-triazine and the 1,2,3-triazole moieties in chelating metals. Compounds with general structure **46** [110] were moderate inhibitors of the BACE-1 enzyme, with the most active compound having a phthalimide pendant and anIC_50_ = 2.2 *μ*M. They showed some potential as neuroprotective agents, increasing the % of PC12 cell viability treated with A*β*_25-35_ and displaying no cytotoxic effect. The 4-bromophenyl-substituted analogue also had an acceptable ability to chelate Fe^2+^ with a 3 : 2 complex formation with the metal.

In the series of triazine-based compounds [111], an interesting set of analogues was reported, bearing di-(thiophene-2-yl) substitutions and different aryl hydrazone moieties (**47**, Figure 15). Inspired by other previously reported cyclic guanidine MTDLs, the authors exploited the use of the thiophene rings to modulate the lipophilic characteristics of the compounds and to increase the interactions within the BACE-1 active site, while varying the aryl pendants linked to the hydrazone functionality in the search for antioxidant and radical scavenging potential. The series had good to moderate inhibitory activity against BACE-1, and after a nice evaluation of the SAR around the aryl pendants, the 2-indole-substituted analogue resulted in the most potent inhibitor with an IC_50_ = 0.69 *μ*M. Also the hydroxylphenyl-substituted compounds were of interest, especially for the higher scavenging potential displayed in the DPPH assay (IC_50_ = 7 *μ*M, compared to quercetin, whose IC_50_ value is 4.6 *μ*M). The abovementioned 2-indole-substituted analogue resulted to be noncytotoxic in the PC12 neuronal cells in concentrations of up to 10 *μ*M and was selected for testing the metal-chelating activity, showing the ability to chelate Zn^2+^, Fe^2+/3+^, and Cu^2+^in different stoichiometries.

As additional modifications to the triazine core, compounds with general structure **48** (Figure 15) were reported [112]. Here, the introduction of the aryl phenoxymethyl-1,2,3-triazole moiety added further potential to display metal-chelating and antioxidant effects. In these hybrids, substituted phenyl groups replaced the two thiophene rings and the pendant aryl attached to the hydrazone functionality was also O-linked to the 1,2,3-triazole group. The compounds were tested for their ability to inhibit BACE-1, and when the Ar group was a propylisoindoline fragment, the highest potency was achieved, corresponding to an IC_50_ of 18 *μ*M (67.09% inhibition at 30 *μ*M). These tool compounds were evaluated in the DPPH and MTT assays, showing only mild activity as antioxidants and a moderate neuroprotective activity in PC12 cells treated with A*β*_25-35_. However, the compound containing a pendant 4-nitrobenzyl group showed a higher antioxidant effect and was also able to chelate Fe^2+^ and Zn^2+^ in a 1 : 1 stoichiometry.

These interesting series of compounds, combining different pharmacophoric elements, need to undergo further optimization but are certainly of interest as MTDLs able to address some of the most common hallmarks of AD.

**Figure 16:**
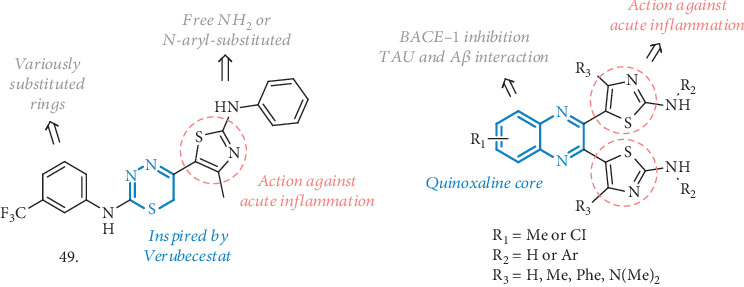
BACE-1 inhibitors with anti-inflammatory activity related to COX interaction.

BACE-1 inhibitors able to interfere with the inflammation process arose from a thiazolyl-thiadiazine scaffold, which Sagar et al. used to embark with the synthesis of novel MTDLs for AD (**49**, Figure 16) [113]. They were inspired by Verubecestat [114] and other thiadiazine-1,1-dioxide scaffolds acting as BACE-1 inhibitors, along with thiazole-based compounds useful in reducing acute inflammation status, thus potentially able to interfere with COX activity.

The prototypic compound **49** (bearing the CF_3_ substituent) was the most potent BACE-1 inhibitor of the series, with an IC_50_ of 9 *μ*M, and it was also evaluated in animal models of carrageenan-induced acute inflammation. There was a high percentage of edema inhibition (70%), comparable to the treatment with Diclofenac, while the effects were slightly less marked on chronic inflammation induced by Formalin (58%), compared to Celecoxib as the reference. The memory-enhancing effect was assessed in an AlCl_3_-induced AD rat model, where a significant and robust improvement in behavioral tests, such as the elevated plus maze or the Y-maze was observed. AlCl_3_-treated animals also showed a significant increase in MDA levels, a marker for lipid peroxidation and oxidative stress. Notably, MDA levels were reduced in treated rats while SOD activity was increased, thus suggesting an antioxidant ability of the compound. No detrimental effects were observed on hematological parameters, and the healthy hippocampus region was the most convincing proof of the protection from neuronal cell degeneration induced by the treatment. Moreover, no hemorrhagic damage or lesion was reported in the stomach and intestine of the animals, demonstrating high gastrointestinal safety. This compound represents a valuable example of how the BACE-1 inhibition and anti-inflammatory activity combined together could serve as efficient agents against AD.

Another nice example of quinoxaline-based molecules acting as BACE-1 inhibitors and useful to modulate inflammation has been reported by the same authors (**50**, Figure 16) [115]. The new compounds were rationally designed to interact with BACE-1 over BACE-2 and to increase binding affinity for COX-1 and COX-2 enzymes, thanks to the introduction of thiazole rings that could establish H-bonds with these enzymes' residues. All the analogues inhibited BACE-1 in the micromolar range, especially those with the unsubstituted amino functionality (R_2_=H). The acute and chronic anti-inflammatory effects were evaluated in carrageenan- and formalin-induced rat paw edema studies, respectively. At doses of 50 mg/kg, the best performing compound showed an inhibition of the edema up to 69% and 55% in the two animal models, even though no *in vitro* inhibition of COX-1 or COX-2 was measured. Having the required activity profile, the compounds were evaluated *in vivo* in behavioral tests, such as the Y-maze, conditioned avoidance response, and elevated plus maze. In all the tests, there were significant effects with reduction of the amnesic effect aroused from AlCl_3_ injections, significant decrease in conditioned avoidance response, and improvement in spatial working memory. Additional antioxidant activity and a promising free radical scavenging effect were confirmed by lipid peroxidase (LPO) and SOD assays in rat brains, with a reduction in MDA levels compared to the control. For their potential as COX inhibitors, the safety on the gastrointestinal tract was checked, evidencing no damages but a minimal lesion in the gastric section. Merged together, BACE-1 inhibition and anti-inflammatory activity can promote beneficial effects on AD and the concomitant antioxidant and antiamyloid potentials observed *in vivo* made these structures interesting MTDLs.

### 2.11. The Multitarget Effect on AD Not Related to ChE or BACE-1 Inhibition

A slightly different approach, aimed at targeting A*β*-mediated toxicity and self- and metal-induced aggregation, together with oxidative stress and no enzymatic activity against any of the common AD-related systems, may offer a great opportunity for the discovery of neuroprotective MTDLs.

Histone deacetylases (HDACs) have attracted attention for their roles in AD brains. HDACs are a class of enzymes that catalyzes the removal of acetyl groups from the lysine residues of both histone and nonhistone proteins [116]. The 18 existing isoforms can use either a zinc- or a NAD^+^-dependent mechanism to accomplish the deacetylation process. Classes I (isoforms 1, 2, 3, and 8), IIa (isoforms 4, 5, 7, and 9), IIb (isoforms 6 and 10), and IV (isoform 11) are zinc-dependent metalloamidases, while Class III HDACs (sirtuine) are the NAD-dependent enzymes. Several inhibitors of selected isoforms have already been successfully tested as promising anticancer agents [117–118]. Nevertheless, the inhibition of HDACs can also provide neuroprotection and enhance synaptic plasticity as well as learning and memory, thus representing a valuable approach for AD treatment [119]. In particular, HDAC2 and HDAC3 have a critical role in controlling memory-related genes [120], while dampening HDAC6 activity enhances pTAU and A*β* clearance [121–122]. Moreover, HDAC2 and HDAC6 seem to be overexpressed in the cortex and hippocampus of AD patients [123].

**Figure 17:**
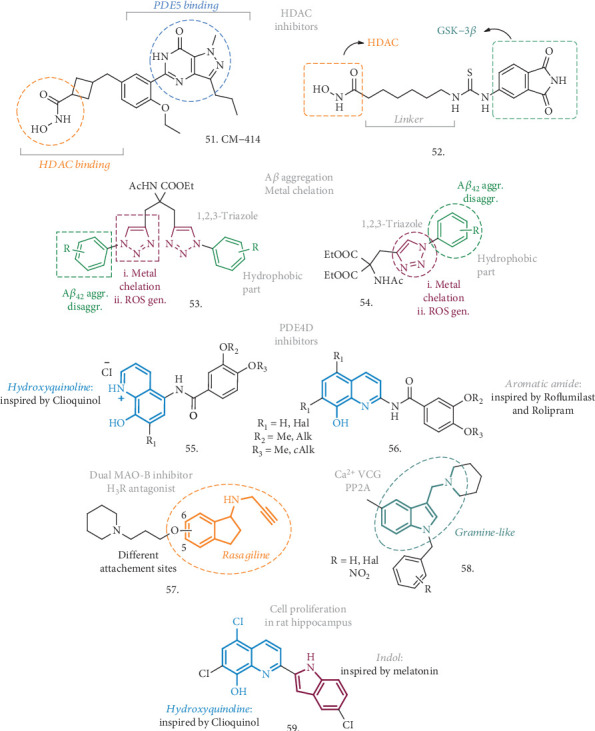
General structures of novel MTDLs to treat AD whose actions are not related to their activities on ChEs or BACE-1 enzymes.

With the aim of combining the effect on HDACs and other AD-related proteins, Cuadrado-Tejedor et al. have explored the effect of a concomitant inhibitor of HDAC6 and PDE5, namely compound **51** (CM-414, Figure 17) [124]. Previously, a cocktail of two different drugs acting on these two enzymes (Vorinostat and Tadalafil) gave *in vivo* positive effects, by alleviating cognitive deficits in AD mice and by reversing the reduced density of hippocampal neurons [125]. In this case, a single compound is responsible for the dual activity, with a moderate class I HDAC activity and a potent inhibition of HDAC6 and PDE5. Compound **51** is a pyrazolopyrimidinone that was inspired by known HDAC and PDE5 inhibitors and bears some key elements endowing it with the essential pharmacophoric features. It also possesses favorable ADME properties and a safe profile in terms of toxicity and cardiovascular safety. CM-414 has IC_50_ against HDAC1-3, HDAC6, and PDE5 of 310, 490, 322, 91, and 60 nM, respectively, with the hydroxamic acid moiety being responsible for the HDAC activity. The synergic effect of HDAC/PDE5 inhibition is responsible for an increase of histone 3 lysine 9 (AcH3K9) acetylation in WT neurons at 10 nM, which correlates with the same effect on SH-SY5Y starting from 64 nM. In Tg2576 neurons, similar effects were observed at 100 nM, where also the effect on hAPP processing and pTAU were evaluated, highlighting a decrease in A*β*_42_ precursors and pTAU levels. When preincubated with hippocampal slices (200 nM), compound **51** rescued the synaptic plasticity impairment in APP/PS1 AD mice, with synaptic potentiation. After the evaluation of PK parameters, toxicity, and BBB penetration in the in vitro PAMPA assay, this analogue was tested in Tg2576 mice, choosing the dose of 40 mg/kg as the optimal one for having acceptable brain concentration and half-life. After 2 weeks of treatment, compound **51** was able to rescue the memory impairment in the fear conditioning (FC) test, with a freezing response similar to WT mice, while a 3-week treatment followed by the Morris water maze test demonstrated a positive effect on spatial memory. To further explain these activities, the authors found that soluble A*β*_42_ and pTAU levels decreased in treated mice (but not in WT ones), paralleled by an increase of the inactive form of GSK-3*β*. Moreover, **51** increased the spine density on apical CA1 dendrites, upregulated markers of synaptic plasticity, and induced the restoration of some of the downregulated genes in Tg2576. A 4-week treatment also led to an enrichment of gene expression and related synaptic transmission in the hippocampal region, and these changes were triggered by an epigenetic mode of action. Overall, compound **51** represents an optimal starting point in the discovery of novel HDACs and PDE5 inhibitors as novel and promising agents to treat AD-related dysfunctions.

To a similar extent, De Simone et al. have recently proposed a series of HDAC/GSK-3*β* inhibitors here represented by compound **52** (Figure 17) [126]. As already mentioned, GSK-3*β* plays a central role in the pathogenic mechanisms of AD through the phosphorylation of pTAU, and the close connection between the latter and HDACs has already emerged. For instance, the neurotoxicity induced by HDAC1 has already been linked to GSK-3*β* activity [127], while the enhanced phosphorylation of HDAC6 by GSK-3*β* has been connected with an increase in the activity of this HDAC and pTAU phosphorylation [128]. Compound **52** is a combination of pharmacophoric elements where the HDAC-interacting part is once again represented by hydroxamic acid while the phthalimide-like scaffold served as the binder for the ATP-site of GSK-3*β*. This analogue is able to inhibit HDAC1, HDAC6, and GSK-3*β* in the low micromolar range of concentration (12.78, 3.19, and 2.69 *μ*M, respectively). SH-SY5Y cells were used to determine the effect of this compound *in vitro*, by analyzing the level of acetylation of tubulin and histone H3 at lysins 9 and 14, and the phosphorylation of pTAU. Treated cells showed hyperacetylated *α*-tubulin, while no effect was observed on AcH3K9 or K14, highlighting a preferential action through HDAC6; pTAU hyperphosphorylation induced by copper was counteracted by 10 *μ*M concentrations of **52**. Despite the fact that HDAC inhibitors are used as anticancer agents, the molecule was safe in this cell line up to 100 *μ*M, and it was also able to efficiently contrast H_2_O_2_-induced oxidative stress, with an effect also on the levels of p53 expression. Moreover, compound **52** was able to promote neurogenesis, as confirmed by the induced expression of recognized markers of neurogenesis such as GAP43, N-myc, and MAP-2, and it had an immunomodulatory activity on microglia, producing a shift from neurotoxic to neuroprotective phenotype. Starting from 50 *μ*M, a clear effect on zebrafish development was also observed and correlated to the inhibition of GSK-3*β*. Although additional studies have to be assessed, the profile of compound **52**, coupled with its low MW and high solubility, make it a promising hit compound for the development of innovative AD-modifying agents.

Kaur et al. identified two series of triazole-based compounds (**53** and **54**, Figure 17) which are able to address four of the major AD hallmarks, including A*β* aggregation, metal-induced A*β* aggregation, metal dyshomeostasis, and oxidative stress [129, 130]. These analogues resulted from the combination of a hydrophobic part, namely the substituted phenyl rings, which are responsible for the contacts with the A*β* peptide and the antiaggregation effect, and a metal chelator part, involving the ditriazole moiety, able to modulate copper-mediated A*β*_42_ aggregation and reduce the oxidative stress.

Amongst the triazole-bearing compounds with general structure **53**, the R=*o*-CF_3_ substituted analogue was the best performer of the series and displayed the most potent inhibitory activity against self-mediated A*β*_42_ aggregation in the ThT fluorescence binding assay, with an IC_50_ of 8.065 *μ*M (even better than the reference curcumin). The significantly reduced formation of A*β*_42_ fibrils was also confirmed by TEM assay. Moreover, the coincubation of preformed fibrils with a 40 *μ*M solution of this agent for 24 h had the capability of reducing the amount of A*β* fibrils in the TEM assay, highlighting a disruptive effect on preformed A*β*_42_ fibrils. The compound also showed metal-chelating ability, as assessed by UV-Vis spectroscopy, thus inhibiting Cu^2+^-induced A*β*_42_ aggregation and promoting the disaggregation of Cu^2+^-induced A*β*_42_ fibrils at a concentration of 40 *μ*M. Furthermore, the multifunctional ligand influenced the generation of ROS by preventing the copper redox cycle in a Cu-ascorbate redox system. The compound was not cytotoxic and did not influence the viability in SH-SY5Y cells in concentrations of up to 50 *μ*M. Noteworthy, it was also able to inhibit the toxicity induced by A*β*_42_ aggregates in the same neuronal cell line. Taken together, all these activities make this compound a promising neuroprotective candidate, worthy of further *in vitro*/*in vivo* investigation.

The use of diethyl acetamidomalonate to build up a 1,2,3-triazole core (the metal-chelating part), *N*-linked to the substituted phenyl groups (the hydrophobic part), led to compounds with general structure **54**, which were meant to interact with the hydrophobic pocket of A*β* aggregates. The 2-iodophenyl group was the best performing in the ThT assay, inhibiting A*β*_42_ self-aggregation by 78% with an IC_50_ = 4.6 *μ*M, as also confirmed by TEM analysis. In addition, this analogue was able to disaggregate preformed fibrils at a concentration of 20 *μ*M. When tested for its metal-chelating characteristic, it resulted in being able to complex Cu^2+^ in a 2 : 1 stoichiometry and this maintained the metal in a redox-inert state preventing the production of ROS. Also the effect on Cu-mediated A*β* aggregation was evaluated, and while the presence of Cu alone increased the formation of A*β*_42_ fibrils, the treatment with the selected compound was followed by its drastic decrease. The same disruptive effect was observed on preformed fibrils whose precipitation was induced by the presence of the metal. The compound had no effect on SH-SY5Y neuronal cell viability whilst it could increase the same viability by up to 78% at 50 *μ*M after A*β*-induced toxic insult. Hence, there is plenty of evidences to state that these structures could substantially contribute to the discovery of novel MTDLs, acting on the principal hallmarks of AD and thus endowed with neuroprotective effects.

A nice example of PDE inhibitors coupled with a multifaceted activity against A*β*-induced toxicity and metal-chelating/antioxidant properties came from the work of Hu et al. (**55** and **56**, Figure 17) [131]. They proposed hybrid compounds merging the metal ion chelating framework of chloroquine and the key binding site fragments of the known PDE4 inhibitors Rolipram and Roflumilast, already tested in preclinical models of AD [132] (see also NCT01433666 and NCT02051335). The result was a series of novel (halo)hydroxyquinolines linked via an amidic bond to di-alkoxy-substituted aryls, which resulted to be a *μ*M inhibitor of PDE4D. Differently from the reference compounds, these analogues exerted a good antioxidant activity in the ORAC assay (comparable to Clioquinol) and showed the ability to chelate metals, including Cu^2+^, Zn^2+^, and Fe^2+/3+^, with a stoichiometry of 2 : 1 ligand : metal in the case of Cu^2+^. The PAMPA test assessed the potential of these analogues to be brain penetrant and cross the BBB, thus prompting the author to perform further analysis. In the Cu^2+^-ascorbate system, no significant production of ROS was observed after coincubation with the compounds, preventing Cu redox by metal chelation. A similar effect was observed in SH-SY5Y cells where besides the lack of a cytotoxic effect, there was a concentration-dependent protective effect against BuOOH-induced intracellular oxidative stress. The effects on metal-induced A*β* aggregation were evaluated, and both the ThT and TEM analysis confirmed that title compounds influenced Cu-induced aggregation. Being safe up to 2000 mg/kg dose and relatively stable in liver microsomes, some compounds were also tested *in vivo* in the Morris water maze test, performed with mice affected by A*β*_25-35_-induced cognitive dysfunction. Compared to the reference PDE inhibitors, the results indicated an enhancement in cognitive spatial memory and behavioral performances, in addition to a protecting effect on hippocampal neurons. Clearly, these structures represent promising candidates for the development of a novel class of anti-AD agents.

As already discussed, the combination of the cholinesterase inhibitory activity with the MAO inhibition may represent an attractive approach to treat some neurodegenerative diseases, such as PD or AD. In a similar manner, the combination of MAO inhibitors with histamine receptor H_3_R antagonists may open up the way for the development of interesting MTDLs [133]. To this extent, two compounds arising from the combination of the 3-piperidinopropyloxy moiety with Rasagiline (MAO inhibitor) have been proposed by Stark et al. (**57**, Figure 17), with the only structural difference represented by the anchoring point on the aminoindane skeleton. The two analogues showed low nanomolar affinity for H_3_R, and only the 5-linked one had inhibitory activity on MAO-B in the nanomolar range, thus demonstrating a preference for linear vs. branched structures. This compound had also a preference for H_3_R over H_1_R and H_4_R and dopamine D2 and D3 receptor subtypes. Moreover, both of the structures showed low cytotoxicity in neuroblastoma cells and were optimal candidates endowed with drug-like properties. No further evaluations were performed, not even regarding the chiral nature of the compounds and the possible separation of the two enantiomers. However, there is a relevant perspective for these analogues to serve as lead structures for the design of MTDLs useful in the treatment of neurodegenerative diseases.

In the search for agents targeting AD-inducing processes, a new class of compounds acting on Ca^2+^ VGC and preventing the inhibition of phosphatase 2A (PP2A) has been reported (**58**, Figure 17) [134]. As already observed, Ca^2+^ overload due to an altered VGC opening is common in several neurodegenerative diseases [70], but PP2A downregulation has also been linked to the progression of AD [135], since this enzyme plays a role in the phosphorylation of pTAU. Inspired by the structure of natural alkaloid Gramine, whose derivatives have already been evaluated as a potential treatment for neurodegenerative disease [136], Gonzales et al. used the indole core to create a new series of *N*-benzyl-substituted compounds able to prevent PP2A inhibition and Ca^2+^ overload. The preincubation of these analogues with SH-SY5Y cells subjected to high K^+^ concentration prevented the cytosolic increase of Ca^2+^, proving the blockade effect on cell depolarization and VGC opening, and showing IC_50_ values ranging from 1.8 to 4.8 *μ*M. A mild antagonist effect was also reported on NMDA receptors in rat cortical embryonic neurons, even if this may be ascribed to an indirect interaction with PP2A. In fact, this latter can form stable complexes with NMDA receptors [137], leading to receptor dephosphorylation and desensitization, with a lower influx of Ca^2+^. To demonstrate this hypothesis, PP2A activity in SH-SY5Y cells treated with okadaic acid (a known PP2A inhibitor) was evaluated, as this represent a common AD model to study PP2A dysfunction and to assess the effects of the new compounds on restoring the enzyme activity. The loss in PP2A activity was prevented by pre- and coincubation with most of the compounds at 0.1 *μ*M, confirming their ability to act as PP2A-activating drugs. At the same time, okadaic acid had a detrimental effect on cell viability and the treatment with these agents increased SH-SY5Y cell viability up to an extent of 70%, resulting nontoxic *per se* at concentrations 30-fold higher than the one necessary to induce neuroprotection. All these results confirmed how this pharmacological combination could represent a valuable tool to address AD-related dementia.

A very detailed research involving several quinoline-indole-based derivatives pointed out the ability of these compounds in promoting cell proliferation in the adult murine hippocampus, as a result of their neuroprotective effect against A*β*-related toxicity and oxidative stress [138]. Although there would be more than one analogue that may be worthy of investigation, the optimized compound **59** (Figure 17) has been identified as the most promising candidate of the series and arose from the combination of the Clioquinol structure with the substituted indole fragment. The ORAC-FL test revealed that the compound had higher antioxidant efficacy (ORAC value = 5.0) compared to Clioquinol, and the PAMPA assay confirmed the potential to be BBB penetrant by passive diffusion. In the ThT assay for A*β*_1-42_ self-induced aggregation, it showed higher inhibitory activity than parent and reference compounds and it had a significant effect in disaggregating preformed fibrils. The new derivative was able to chelate Cu^2+^, Zn^2+^, Fe^2+^, and Fe^3+^ generating Cu^2+^/compound complexes in a 1 : 2 stoichiometry. Following this chelating potential, the effect on metal-induced A*β* aggregation was also evaluated, demonstrating a substantial rate of inhibition for Cu^2+^-associated A*β*-aggregation and disaggregation of Cu^2+^-promoted preformed fibrils. The compound had no neurotoxicity in the PC12 cell line in concentrations of up to 50 *μ*M, but rather revealed the ability to increase the number of cells, possibly via a MAPK-dependent mechanism. There was also a clear effect in reducing the production of ROS in SH-SY5Y stimulated cells. The HCl salt formulation displayed a considerable metabolic stability in liver microsomes (*T*_1/2_ = 116 min), and no acute toxicity or significant alteration was observed in adult C57BL/6 mice (up to 2000 mg/kg). In the same animal model, following ICV injection of the hydrochloride salt, there was a clear effect in inducing hippocampal cell proliferation from the reservoirs of neuronal stem cells in the subgranular zone, and from the neural precursor in other hippocampus regions, as confirmed by immunohistochemical analysis of the mice brains. Double transgenic APP/PS1 mice (a model of AD) were used to assess the cognitive and memory strengthening effect in the Morris water maze test, where the treatment with the compound demonstrated a significant amelioration of memory impairment and cognitive dysfunctions. Daily doses of 30 mg/kg were safe and well tolerated, and the chronic oral treatment also lead to a remarkable reduction in A*β* plaque deposition, in correlation with the positive effects on learning and memory. In conclusion, this analogue is a nice example of an MTDL with optimal *in vitro* properties and proven *in vivo* activity which makes it an attractive agent to be further pursued for AD treatment.

### 2.12. The Role of Synthetic Peptides in AD

Although the use of small molecules as MTDLs is the most common approach towards the discovery of new treatments for AD, it may be interesting to spend a few words on the role of synthetic peptides as novel agents against AD. A*β* oligomers, protofibrils, and prefibrillar aggregates are pivotal players in the toxic insult leading to brain damage. Other neurodegenerative disorders, such as ALS, prior disease, or HD, are characterized by the aggregation of misfolded proteins. Synthetic peptides offer the possibility to modulate this process by interacting with endogenous peptides or interfering with protein aggregation. In the past decades, the use of peptide-based drugs has emerged as a valuable tool to treat different pathologies [139–141] and increasing interest has been paid to their effects as disease-modifying tools. They can also serve as probes and diagnostic tools for an early diagnosis or discovery of neurodegeneration [142]. Potential targets for peptide-based therapies could be the cognitive impairment (e.g., neuroprotective effect of insulin) or the modulation of A*β* formation and aggregation through protein-protein-like interactions. These peptides can be either natural or modified A*β* derivatives, capable of interfering with the A*β* structure and aggregates, and/or bearing nonnatural amino acids, which have already proven their ability of inhibiting A*β* aggregation and attenuating mild cognitive impairments [143]. Nevertheless, these agents have to penetrate in the brain; thus, they have to be evaluated for their ability to cross BBB and reach their targets. Interestingly, some oligopeptides have already shown significant effects in animal models of dementia [144] and an even narrowed set has been progressed into clinical studies [143]. Despite the fact that they may not perfectly fall under the definition of MTDLs, synthetic peptides have the potential to hit or modulate multiple targets associated with AD dysfunctions, and thanks to their versatility, they can also serve as disease-modifying agents, giving new perspectives and opportunities in the search for novel AD treatments.

## 3. Conclusions

The multifaceted and complex nature of AD has triggered tremendous research efforts towards developing multitarget therapeutic strategies. These strategies are mainly developed to target multiple factors involved in the progression of AD such as mitochondrial dysfunction, deposition of amyloid aggregates in the brain, oxidative stress, and altered brain glucose metabolism. This review focused on the development of small molecules that were rationally designed to interact with multiple targets directly related to the etiology of AD. In addition, structural modification of the lead compounds enabled introduction of key features such as metal chelation and antioxidant and anti-inflammatory activities which have proven to be beneficial properties in the identification of potential therapeutics of AD. The design of dual inhibitors of enzymes involved in the progression of AD is one of the most common strategies implicated in the development of MTDLs. Inhibitors of ChEs have been widely used in the design of dual enzyme inhibitors mainly through molecular hybridization. The majority of research in this area focused on using scaffolds of AD drugs such as THA and Donepezil with reported ChE inhibitory activity. Hybridization with scaffolds with known inhibitory activities against GSK-3*β*, MAOs, PARP-1, and PDEs have resulted in multiple examples of MTDLs. Similarly, hybridization of ChEs with NMDA receptor antagonists as well as 5-HT_6_R antagonists evolved as a promising approach for the development of MTDLs. BACE-1-centered MTDLs have received growing attention from researchers in the previous years. Numerous MTDLs have been reported that combine BACE-1 inhibition with anti-inflammatory and antioxidant properties.

The unfavorable pharmacokinetic properties of reported MTDLs remain to be the main barrier for further clinical translation of these compounds. Thus, efforts focusing on optimizing oral bioavailability, metabolic clearance, and CNS permeation of lead compounds would be critical in advancing more MTDLs into clinical trials. Moreover, MTDLs that are designed to target different pathological cascades of AD are more likely to be efficient against multifactorial AD in comparison to MTDLs designed against a single pathway of AD progression.
